# Validation and cross-cultural adaptation of the six-dimension scale of nursing performance- arabic version

**DOI:** 10.1186/s12912-024-01740-3

**Published:** 2024-01-19

**Authors:** Wafa’a F. Ta’an, Jehad A. Rababah, Mohammed M. Al-Hammouri, Jumana Yousef, Tareq Lewis Mukattash, Brett Williams

**Affiliations:** 1 Community and Mental Health Nursing Department, Faculty of Nursing, Jordan University of Science and Technology, P.O. Box 3030, Irbid, 22110 Jordan; 2 Department of Adult Health Nursing, Faculty of Nursing, Jordan University of Science and Technology, Irbid, Jordan; 3 Department of Clinical Pharmacy, Faculty of Pharmacy, Jordan University of Science and Technology, Irbid, Jordan; 4 Department of Paramedicine, Monash University, Clayton, VIC Australia

**Keywords:** Psychometric Properties, Arabic Version, Six-dimension scale, Nursing, Job performance

## Abstract

**Background:**

Nursing performance is a key indicator of patients’ care quality and safety. Most healthcare research tools are available in the English language; however, nurses around the world can employ these tools if rigorously adapted and cross-culturally validated.

**Aims and objectives:**

This study aims to provide a cross-cultural adaptation and validation of the six-dimension scale of nursing performance to be used among Arabic-speaking nurses.

**Design:**

The study employed a descriptive, correlational design with a cross-sectional approach.

**Methods:**

A five-step cross-cultural adaptation process was adopted. The scale was administered to 216 Jordanian nurses between January 2022 to April 2022. SPSS and AMOS were used for descriptive and correlation analyses and testing the six-dimension model through structural equation modeling (SEM).

**Results:**

The current study produced a valid, reliable, culturally adapted Arabic language version of the six-dimension scale of nursing performance. The internal consistency of the tool was supported by a Cronbach Alpha’s value of 0.99. The model’s goodness of fit indices were: CFI = 0.96, RMSEA = 0.048, and CMIN/df = 1.49. The exploratory factor analysis (EFA) of the scale identified three factors with eigenvalues greater than 1.00., explaining 75.22% of the variance. A subsequent EFA, specifying six factors, yielded 79.79% explained variance. All item factor loadings exceeded 0.30, confirming the scale’s robust factor structure.

**Conclusions:**

This study proved that following a robust cross-cultural adaptation process results in a reliable and valid measure of nursing performance to be used among Arabic-speaking nurses. The study supports the dimensionality of nursing performance as evidenced by the SEM results. Therefore, the findings have the potential to considerably enhance studying nursing performance in healthcare fields in Arabic-speaking nurses.

**Relevance to clinical practice:**

The validation and cross-cultural adaptation of the Arabic version of the Six-Dimension Scale of Nursing Performance have direct implications for improving the quality of nursing services, enhancing patient safety, promoting cultural competence, and supporting the professional growth of Arabic-speaking nurses.

## Introduction

Performance is considered “the ultimate dependent variable in human resource management” [1]. In healthcare, nursing performance is crucial due to its correlation with patient safety, and care quality, in addition to its impact on financial and other organizational outcomes. Healthcare organizations cannot improve or transform without the excellent performance of their employees [2]. The literature review of Krijgsheld et al. illustrated the importance of nursing performance in being multi-dimensional which is conceptualized as task performance, adaptive performance, counterproductive work behavior, or contextual performance [2].

Similar to all businesses, healthcare institutions seek to achieve their goals and planned outcomes. Healthcare institutions share a common goal of enhancing the quality of care and improving patients’ outcomes and satisfaction [3]. This can be achieved by successfully leading the members of an organization to their optimal level of performance [4]. The performance of the nurses is significantly valuable to the success of the healthcare system as they comprise the largest workforce group in most healthcare institutions. Additionally, literature shows that nurses spend 70% of their time in direct patient care [5]. Therefore, they play a significant role in organizational achievements in all healthcare settings.

Schwirian defined nursing performance as the extent to which a nurse performs his or her roles and responsibilities to achieve high-quality healthcare services [6]. This perspective demonstrates both task performance and contextual performance as it represents more than just the core job responsibilities of a nurse (task performance) but extends to the discretionary extra-role behaviors exemplified by interpersonal and leadership behaviors (contextual performance) and the adaptive behaviors in which a nurse responds to challenges such as the ability to work in challenging situations [7].

The necessity for a valid tool that operationalized ‘nursing performance’ was the driving force for the creation of the six-dimension scale of nursing performance [6]. The tool can be used as self-appraisal or supervisor/educator appraisal of performance. It consists of 52 items categorized into six subscales: leadership (L), critical care (CC), teaching/collaboration (TC), planning/evaluation (PE), interpersonal relations/communications (IC), and professional development (PD) comprising 5, 7, 11, 7, 12, and 10 items, respectively. The scale can be used to assess nursing performance in terms of frequency or quality. Responses for all items can be rated over a four-point Likert scale. The six subscales’ items are arranged in random order, and the total subscale score can be calculated by calculating the mean of the items for each subscale. Cronbach’s alphas of subscales of the Six-dimension scale of nursing performance ranged from 0.84 − 0.97 which reflects excellent reliability [6]. In a previous study that used this tool, the reliability coefficient of the scale was 0.96, and the subscales ranged between 0.78 and 0.90 indicating excellent internal consistency [8].

Nursing research is rapidly expanding horizontally (widening the scope and geographically), vertically (depth and complexity), and diagonally with multiculturality and interdisciplinary approaches. With such an increase in research, there is a need to avail tools for researchers to overcome the lingual and intercultural challenges. Most research tools are available in the English language; however, people around the world can employ these tools if rigorously adapted and validated cross-culturally. Among these tools is the six-dimension scale of nursing performance which measures performance among nurses [6]. It is the most commonly used tool in studying performance among nurses [9, 10]. Yet, there is no validation for an Arabic version of this tool.

To enhance the usability and credibility of the six-dimensional nursing performance scale for Arabic-speaking nurses, the development of a valid and reliable Arabic version of the tool is imperative This study aimed to examine the psychometric properties of the Arabic version of the six-dimension scale of nursing performance, knowing that Arabic is the third most spoken first language in the world [11]. Establishing a valid and reliable tool that is understandable by participants will enhance the comparability and generalizability of the research findings.

## Methods

### Design, setting, and sampling

The study implemented a cross-sectional design to study the validity of the six-dimension scale of nursing performance among Jordanian nurses. The cross-sectional design was used because of its compatibility with the purpose of the study. It can test associations among study factors and provide evidence for the relationships [12]. This research was planned to investigate the six-dimension scale of nursing performance dimensionality and its validity in measuring the concept of nursing performance. Thus, the cross-sectional design was utilized to compare the study findings and evaluate the relationships between the instrument’s subscales at the same time.

Four hospitals in the Northern and Middle regions of Jordan were selected for this study in a convenient approach. To ensure the study’s broader applicability, we deliberately chose hospitals that belonged to different sectors within the Jordanian healthcare system: governmental, private, non-profit, and educational (university-affiliated) hospitals. Inclusion criteria for hospital selection required a minimum patient capacity of one hundred.

Data collection was performed during the time between January 2022 and April 2022. Nurses were invited to participate in the study if they complied with the inclusion criteria that included elements to ensure that the study would produce the needed data for achieving reliable and reproducible results. The criteria were any full-time employed nurse with a minimum experience of six months. This criterion was used to ensure that nurses have sufficient exposure to various nursing practices within their scopes.

By the end of the data collection period, 216 nurses participated in the study. According to the guideline of Hair et al., a range of 100–400 participants composes an adequate sample for structural equation modeling, more specifically, for maximum likelihood estimation [13]. Therefore, the collected data was considered sufficient to start the analysis.

Approval was obtained from the designated institutional review board (IRB reference number: 381/2022) then the data collection process started. Each participant had the time to read the letter of information, sign the consent form, and complete the questionnaire. Participants were recruited from governmental and private hospitals to enhance generalizability.

### Measures

The questionnaire includes two parts; the first includes demographic questions such as educational level, gender, and age. The second part is the six-dimension scale of nursing performance [6]. This tool is a six-subscale instrument that has 52 items. The subscales are leadership (L, 5 items), critical care (CC, 7 items), teaching/ collaboration (TC, 11 items), planning/ evaluation (PE, 7 items), interpersonal relations/ communications (IC, 12 items), and professional development (PD, 10 items). The original English scale was tested for validity and reliability and was found valid and reliable with a Cronbach alpha ranging from 0.84 to 0.97 for the seven subscales [6].

### Translation and cross-cultural adaptation process

Approval to use, translate, and modify the tool was obtained from the original author. To ensure cross-cultural validation, the protocol of Koopmans et al. was followed [14]. The protocol consists of five stages of translation and cross-cultural adaptation. The first step was the forward translation in which two bilingual experts independently cross-culturally translated the scale from English into Arabic; one translator was informed on the topic (with an excellent nursing research background), and another was uninformed (naïve about the scale) with a medical background. The second step was the synthesis, in which one of the study researchers met with the two translators, discussed uncertainties, and reached a consensus on an agreed-upon Arabic version of the scale. In the third step, back translation, two additional bilingual translators, both with expertise in the medical field (a researcher and a Ph.D. student), conducted translations back into English. The fourth step was the expert committee review, in which all translated documents were integrated into a prefinal version of the Arabic questionnaire. The committee consisted of all translators and two researchers. The committee members discussed the semantics and conceptual integrity of the translated questionnaire and assessed its content validity. as well as the cultural adaptability of the translated questionnaire. No issues were found in the language and semantics of the scale. In the final step, pilot-testing, the Arabic version of the six-dimension scale of nursing performance was administered to 35 participants to ensure that the questionnaire was understandable and culturally appropriate. The piloting revealed no concerns about the questionnaire, therefore, the participants in the pilot study were included in the final sample.

### Data analysis

The content and face validity of the scale were assessed, and then SPSS and AMOS were used to analyze the data. Descriptive statistics and frequencies were obtained using SPSS. In addition, SPSS was used to calculate Cronbach’s α values for the nursing performance scale and its subscales as well as the exploratory factor analysis, AMOS was used to carry out the convergent validity, discriminant validity, and confirmatory factor analysis (CFA). The following criteria were used to evaluate the CFA model fitness: (1) Comparative fit index (CFI) of > 0.95, (2) root mean square error of approximation (RMSEA) of ≤ 0.70, and (3) Chi-Square/Degrees of Freedom (CMIN/df) of < 5 [15].

## Results

### Participants’ characteristics and internal consistency

The mean age of the participants was 28.36 (SD = 4.51) years old. On average, the participants had a total experience as a registered nurse of 5.42 (SD = 3.80) years and worked a total of 45.05 (SD = 4.90) hours per week. Female participants represented 53.7% of the study sample. The majority of the participants (90.3%) had a bachelor’s degree in nursing (Table 1).

**Table 1:** Participants’ sociodemographic characteristics

Variable	Mean	SD
Age (years)	28.36	4.51
Experience (years)	5.42	3.80
Work hours/week	45.05	4.90
	Frequency	Percentage
Gender:		
Female	116	53.7
Male	100	46.3
Education:		
Bachelor’s	195	90.3
Master’s	21	9.7
Unit:		
Medical-surgical	76	35.2
Critical care	72	33.3
Pediatrics	28	13.0
Emergency	25	11.6
Other	15	6.9
Marital status:		
Married	109	50.5
Single	107	49.5
Hospital:		
Governmental	53	24.5
Private	51	23.6
University-affiliated	49	22.7
Non-profit	63	29.2

### Content and face validity

Content and face validity were assessed through expert review to ensure that the items in the Six-Dimension Scale of Nursing Performance adequately represented the construct of interest. A total of five experts evaluated the appropriateness and relevance of each item of the Six-Dimension Scale of Nursing Performance. The results of the content validity assessment indicated that the items within the instrument were deemed highly relevant and comprehensive in measuring the intended construct. Expert reviews confirmed that the content adequately represented the domain of interest, affirming the instrument’s content validity.

### Exploratory factor analysis

Using SPSS, exploratory factor analysis (EFA) was performed to get an overall picture of the factors of the Six-Dimension Scale of Nursing Performance. The results showed that there were three factors with eigenvalues greater than 1.00. These factors accounted for 75.22% of the explained variance. EFA was performed again with a pre-determined number of factors (i.e., 6) and the results showed that the total explained variance was 79.79%. Factor loadings of individual items were all above 0.30 providing evidence regarding the factor structure of the Six-Dimension Scale of Nursing Performance subscales.

### Confirmatory factor analysis

**Fig. 1:**
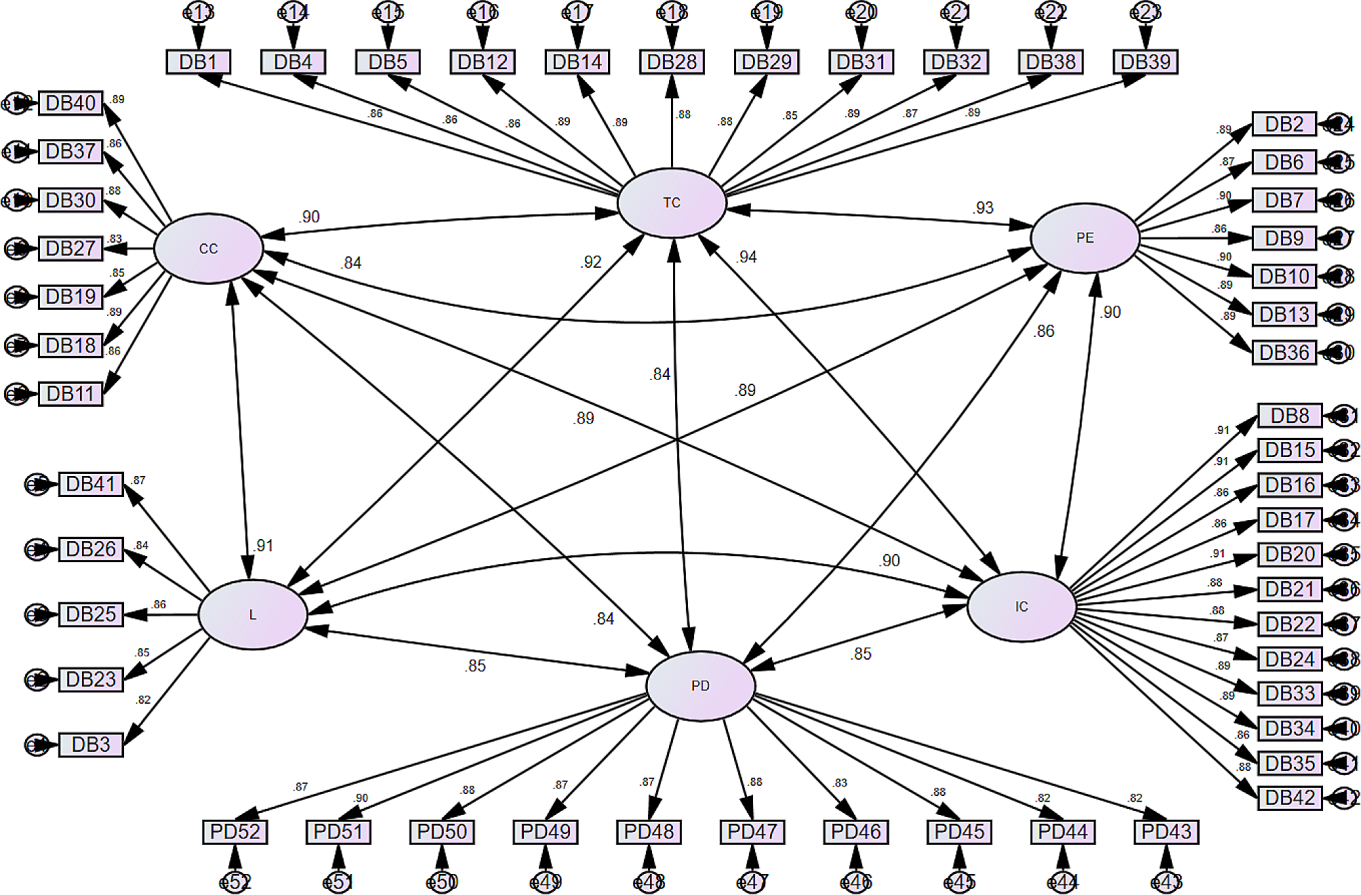
Confirmatory factor analysis

Based on the results of previous literature, a six-factor model was tested. The Chi-square results were as follows: *X*^*2*^ = 1881.52, df = 1259, *p* <.001. The results of the CFA showed that the model meets the criteria for model fitness: CFI = 0.96, RMSEA = 0.048, and CMIN/df = 1.49. In addition, the individual items demonstrated acceptable factor loadings with a range of 0.82 to 0.91. Figure 1 displays the results of the CFA. The final model included a total of 6 subscales. The number of items under each subscale and the values of each item’s factor loadings are presented in Table 2.

**Table 2:** Factor loadings

Subscale: L		Subscale: CC		Subscale: TC	
Item no.	Factor loading	Item no.	Factor loading	Item no.	Factor loading
3	0.82	11	0.86	1	0.86
23	0.85	18	389	4	0.86
25	0.86	19	385	5	0.86
26	0.84	27	0.83	12	0.89
41	0.87	30	0.88	14	0.89
Subscale: IC		37	0.86	28	0.89
8	0.91	40	0.89	29	0.88
15	0.91	Subscale: PD		31	0.85
16	0.86	43	0.82	32	0.89
17	0.86	44	0.82	38	0.87
20	0.91	45	0.88	39	0.89
21	0.88	46	0.83	Subscale: PE	
22	0.88	47	0.88	2	0.89
24	0.87	48	0.88	6	0.87
33	0.89	49	0.87	7	0.90
34	0.89	50	0.88	9	0.86
35	0.86	51	0.90	10	0.90
42	0.88	52	0.87	13	0.90
				36	0.89

### Internal consistency, convergent validity, and discriminant validity

Regarding the internal consistency, the Cronbach’s α of the six subscales were 0.97, 0.96, 0.98, 0.96, 0.93, and 0.97 for the subscales TC, PE, IC, CC, L, and PD respectively. Another indicator of internal consistency was composite reliability (CR) with values of 0.70 and higher support internal consistency. The results of this study showed that the values of CR for the TC, PE, IC, CC, L, and PD subscales were 0.97, 0.96, 0.98, 0.96, 0.93, and 0.97, respectively (Table 3).

**Table 3:** Indicators of internal consistency, convergent validity, and divergent validity

	C. α	CR	AVE	L	CC	TC	PE	IC	PD
L	0.93	0.93	0.72	**0.85**					
CC	0.96	0.96	0.75	0.91	**0.87**				
TC	0.97	0.97	0.77	0.92	0.90	**0.88**			
PE	0.96	0.96	0.79	0.89	0.84	0.93	**0.89**		
IC	0.98	0.98	0.78	0.91	0.89	0.94	0.90	**0.88**	
PD	0.97	0.97	0.74	0.85	0.84	0.84	0.86	0.85	**0.86**

C. α: Cronbach’s α CR Composite reliability, AVE average variance extracted

Note: bold diagonals are the squared root of AVE, and off-diagonals are correlations

Convergent validity of individual subscales was appraised based on an average variance extracted (AVE) of ≥ 0.5. Regarding discriminant validity, the square root of the average variance extracted (AVE) for each construct was computed and compared to the correlations between constructs. The results showed that the AVE values were all above the cutoff point supporting the convergent validity of the six-dimension scale of nursing performance. Discriminant (divergent) validity is affirmed when the square root of AVE for a construct surpasses its correlations with any other construct in the model (Hair et al., 2014). According to the results, as shown in Table 3, the discriminant validity of the six-dimension scale of nursing performance is not supported for all subscales.

## Discussion

The first step in validating a scale in a different language is to ensure that the items on the scale are appropriate for the population being studied [16]. In the cross-cultural adaptation and validating the six-dimension scale of nursing performance, the process includes making sure that the language used is appropriate for the Arabic population, and that the items are relevant to the nursing performance concept that is being measured. Once the items have been determined to be appropriate, the next step is to conduct a validation study for the psychometrics of the Arabic version of the scale. This involves testing the scale on a group of participants who complete the scale and provide their responses on the items. This will provide a better indication of the scale’s validity and reliability. The results of this test should be analyzed to provide scientific evidence that promotes the usage of the scale in future studies.

The current study produced a robust Arabic version of the scale that was ensured through a rigorous cross-cultural translation process by following the protocol of Koopmans et al. that involved the five steps of forward translation, synthesis, back-translation, expert committee review, and pilot-testing [14]. The reliability indices of the scale were found excellent by the evidence of the high Cronbach’s alpha values of the total score as well as the subscales that were between 0.93 and 0.99 as reported in the results section.

Previous studies have explored the validation of nursing performance scales in different languages. For example, a study conducted in Japan found that the nursing performance scale was valid and reliable in the Japanese language with Cronbach alpha values of 0.92 for the total scale and 0.90-0.91 for the subscales [17]. The authors followed a translation process in which two persons forward translated the scale, content validity through a meeting of five nursing researchers and back translation by two other persons.

As the scale is being widely used in nursing research globally, an Iranian study has also used the six-dimension scale of nursing performance [18]. In their study, Jahromi and colleagues reported that the psychometric properties of the nursing performance scale are supportive of its reliability and validity [18]. Due to the absence of a previous Iranian version of the scale, the authors translated it into Persian and back into English through two independent experts. Then ten faculty members were invited to assess the tool’s face and content validities. After that, the authors pilot-tested the translated tool among 30 nursing students and a Cronbach alpha value of 0.97 resulted.

One study in China has reported further analyses [8]. The study intended to measure performance among male nurses in China using Scwirian’s six-dimension scale of nursing performance The authors assessed the reliability of the Chinese version of the scale through the Cronbach reliability coefficient which was 0.96 for the whole scale and 0.78-0.90 for the subscales. They also analyzed the split-half and intra-group correlation for the retest reliability coefficients which were 0.82, and 0.86, respectively.

This research adds value to previous nursing literature and provides opportunities for further utilization of the tested scale. The previously discussed research studies have only briefly reported the translation process in addition to conducting face and content validity, and only used Cronbach alpha to measure reliability. In the current study, the validation process was extended to elaborate on the cross-cultural translation and adaptation process to assure the scale’s validity. Additionally, the analysis was extended to the use of SEM to shed light on the dimensionality of the scale. The confirmatory factor analysis using SEM revealed that the six-dimensional model of the nursing performance scale has good fitness indices when tested on the Arabic version of the scale which strengthens the scientific evidence of the rigor of the six-dimension scale of nursing performance and proves its usability internationally.

## Conclusion

The current study produced a valid, reliable, and culturally appropriate Arabic version of the six-dimension scale of nursing performance, and also, supported the scale’s dimensionality. The process of cross-cultural adaptation yielded a rigorous version of the previously mentioned tool which was demonstrated by the excellent psychometric properties of the Arabic version of the scale. The production of a scale that serves a great scope of nursing leadership practices can be of great benefit to researchers in the field. Future studies investigating nursing performance among nurses can be enhanced using the current study’s findings. Moreover, the study facilitates valid comparisons of study findings across cultures utilizing robust methodological procedures of cross-cultural adaptation. The study can also guide researchers in the process of knowledge translation and transfer in the era of globalizing health research.

### Relevance to clinical practice

This study holds significant relevance for clinical practice in nursing performance assessment, research, quality improvement, and benchmarking. By validating and cross-culturally adapting this scale to the Arabic-speaking context, healthcare institutions can have a reliable tool to assess nursing performance accurately. This allows for better evaluation of nursing staff, identification of areas for improvement, and creating tailored training programs.

At the level of patient care, accurate performance assessment directly impacts the quality of patient care. Nurses who are assessed using a culturally adapted and validated tool are likely to provide more effective and culturally sensitive care, thereby enhancing the overall patient experience and outcomes. Moreover, the availability of a validated Arabic version of the scale contributes to the professional development of Arabic-speaking nurses, allowing them to receive constructive feedback, set goals for improvement, and ultimately advance their nursing skills.

At an administrative level, healthcare administrators and researchers can use the culturally adapted and validated nursing performance scale to conduct studies and benchmark nursing performance across different healthcare institutions. This can lead to the identification of best practices and the implementation of evidence-based strategies to enhance nursing care. Quality assurance processes may also use data gathered through this scale to maintain high standards of care and continually monitor and improve nursing performance in a culturally appropriate manner.

In summary, the validation and cross-cultural adaptation of the Arabic version of the Six-Dimension Scale of Nursing Performance have direct implications for improving the quality of nursing services, enhancing patient safety, promoting cultural competence, and supporting the professional growth of Arabic-speaking nurses.

## Data Availability

The datasets used and/or analyzed during the current study are available from the corresponding author upon reasonable request.
